# Dietary Supplementation with Oregano and Linseed in Autochthonous “Facciuta Lucana” Goats: Effects on Meat Quality Traits in Suckling Kids

**DOI:** 10.3390/ani13193050

**Published:** 2023-09-28

**Authors:** Maria Antonietta Colonna, Despoina Karatosidi, Carlo Cosentino, Pierangelo Freschi, Claudia Carbonara, Francesco Giannico, Caterina Losacco, Vincenzo Tufarelli, Simona Tarricone, Maria Selvaggi, Marco Ragni

**Affiliations:** 1 Department of Soil, Plant and Food Science, University of Bari Aldo Moro, Via Amendola 165/A, 70125 Bari, Italy; mariaantonietta.colonna@uniba.it (M.A.C.); claudia.carbonara1@uniba.it (C.C.); maria.selvaggi@uniba.it (M.S.); marco.ragni@uniba.it (M.R.); 2 Research Institute of Animal Science, Hellenic Agricultural Organization-DIMITRA, 58100 Giannitsa, Greece; despinakaratosidi@elgo.gr; 3 School of Agricultural, Forestry, Food, and Environmental Sciences, University of Basilicata, Viale dell’Ateneo Lucano, 10, 85100 Potenza, Italy; carlo.cosentino@unibas.it (C.C.); pierangelo.freschi@unibas.it (P.F.); 4 Department of Veterinary Medicine, University of Bari Aldo Moro, 70010 Bari, Italy; 5 Department of Precision and Regenerative Medicine and Jonian Area, Section of Veterinary Science and Animal Production, University of Bari Aldo Moro, S.P. per Casamassima km 3, 70010 Valenzano, Italy; caterina.losacco@uniba.it (C.L.); vincenzo.tufarelli@uniba.it (V.T.)

**Keywords:** kids, autochthonous breed, Facciuta Lucana, meat quality, fatty acid profile, sensory quality

## Abstract

**Simple Summary:**

This study evaluated the dietary effects of extruded linseed and oregano on the quality traits and sensory properties of meat obtained from kids of a Lucanian goat population named “Facciuta Lucana”. Thirty-six male kids were divided into three homogeneous groups fed a control feed (C), a diet containing 3% extruded linseed (L), or 3% linseed plus 0.6% oregano (L + O). Meat from the *Longissimus lumborum* muscle obtained from linseed-fed groups showed a lower content of fat and total saturated fatty acids and, in turn, an increase in monounsaturated and polyunsaturated fatty acids, and conjugated linoleic acid, with benefits for human health. Oregano addition to the linseed diet proved to be effective in preserving meat shelf-life, since it lowered the malondialdehyde concentration after 10 days of storage, and improved meat succulence, tenderness, juiciness, and overall acceptance.

**Abstract:**

Extruded linseed (*Linum usitatissimum*) in ruminant diets has been investigated as a strategy to improve the nutritional value and healthiness of meat fat; however, increased polyunsaturated fatty acids may limit the shelf-life of meat. Oregano (*Origanum vulgare*) has a documented antioxidant activity. The aim of the study was to investigate the effects of dietary supplementation with extruded linseed and oregano on goat milk quality and whether the characteristics of goat milk affect the physical and chemical features, fatty acid profile, meat lipid oxidation, and sensory properties of meat from suckling kids. Thirty-six male kids were weaned and divided into three homogeneous groups (*n* = 12); each group was either fed a control diet (C), or a diet containing 3% extruded linseed with or without the addition of 0.6% oregano (“L + O” and “L”, respectively). The diets containing linseed lowered (*p* < 0.05) the saturated fatty acid content in meat, and increased (*p* < 0.05) monounsaturated and polyunsaturated fatty acids and conjugated linoleic acid. Oregano addition to the linseed diet proved to be effective in preserving meat shelf-life, as shown by a significant (*p* < 0.01) reduction in the malondialdehyde concentration after 10 days of storage, and improved meat succulence, tenderness, juiciness, and overall acceptance.

## 1. Introduction

In South Italy, goat farming has been very widespread due to the great ability of goats to adapt to harsh marginal areas. However, in the last decades, many local goat populations have experienced a severe reduction in the number of animals. In the Basilicata region, one of these breeds is the so-called “Facciuta Lucana”, also known by other local names that refer to its most striking feature: two white bands on the muzzle. This breed, according to various testimonies, was present both in family farms consisting of few subjects, as well as in herds of medium proportions, generally not larger than 50 animals. Actually, a total of 95 subjects are present in the whole Basilicata Region (Table S1).

In recent years, an intense research program has been conducted aimed at the recovery and enhancement of the autochthonous sheep and goat populations/breeds of the Basilicata region (Val.Bi.Ovi.Cap. Research project). The preservation of local genotypes is important for rural development and the protection of the environment; furthermore, the association between local breeds and their traditional agri-food products (TAP) represents a great opportunity to convert abandoned marginal areas into sites of cultural interest and food tourism [1].

According to the local traditional rearing system in South Italy marginal areas, kids are raised with their mothers and suckle only dam’s milk until weaning, which generally occurs when they are about 45 days old, in order to use the goat milk for cheese-making. At this age, kids may be slaughtered, providing “capretto” meat, which is greatly appreciated for its organoleptic features [1,2]. 

The low productive performance of these local breeds is the main reason why breeders have turned their interest towards high-production cosmopolitan breeds; therefore, any attempt to rescue local populations must improve the yield and quality of animal products, while respecting local traditions, through effective feeding strategies.

The dietary inclusion of linseed has been widely investigated in livestock animals, showing effects on meat enrichment in unsaturated fatty acids, especially of the n-3 series, such as α-linolenic acid [3,4,5,6]. However, the greater structural instability of unsaturated fatty acids leads meat to increased lipid oxidation, which results in worsened color, flavor, and shelf life [7]. The extent of lipid oxidation may be reduced using natural antioxidants included in the diet, able to delay and limit the occurrence of oxidation processes. Among the natural antioxidants tested in animal feeding, oregano (*Origanum vulgare* L.) is a common essence present in South Italy pastures, the antioxidant activity of which is mainly attributed to carvacrol and thymol [8]. In previous studies, we found that dietary supplementation with extruded linseed and oregano was able to enhance meat fat oxidative stability in Garganica [9], Grigia di Potenza [10], and Jonica breed kids [11]. On the contrary, in an experiment carried out on Gentile di Puglia lambs, the inclusion of oregano in the diet did not provide noticeable effects on meat quality traits [12].

The supplementation of ruminant diets with lipid sources rich in polyunsaturated fatty acids (PUFA) is moderately effective in improving the nutritional value of meat fat due to the biohydrogenation of unsaturated fatty acids, which occurs in the rumen [13]. There is general agreement that during the pre-ruminant stage of life, the fatty acid composition of the muscular and adipose tissues reflects that of the milk consumed, as investigated in lambs [14,15] and kids [16]. Since the nutritional properties of ruminant milk are largely affected by the diet [17,18] and by the season [19], the improvement in meat quality in suckling animals is closely related to the enhancement of the dam’s milk quality [20,21].

The aim of the study was to investigate the effects of dietary supplementation with extruded linseed and oregano on goat milk quality and whether the characteristics of goat milk affect the physical and chemical features, fatty acid profile, meat lipid oxidation, and sensory properties of meat from suckling kids of the autochthonous population named “Facciuta Lucana”.

## 2. Materials and Methods

### 2.1. Animal Management and Diet

All procedures involving animals were ethically and responsibly conducted, according to the Italian government guidelines (Directive 2003/50/EC, received in Italy by D.L. 193/2005) [22].

The study was carried out during February–April 2020 on a dairy farm located in Gorgoglione (in the province of Matera, Basilicata region, Italy; Latitude: 40.3785846, Longitude: 16.1454083, 700 m a.s.l.). 

Two weeks before parturition, in order to allow adaptation to the experimental diets, fifty-one female pluriparous goats of the autochthonous “Facciuta Lucana” breed, homogeneous for weight (52 ± 3 kg) and parity (3–4), were randomly divided into three homogeneous groups (*n* = 17) and fed the following diets: control feed (“C”); a diet containing 3% extruded linseed (*Linum usitatissimum* L., “L”), or 3% linseed plus 0.6% dried oregano inflorescences (*Origanum vulgare* L., “L + O”). The three pelleted feeds were formulated to be isocaloric and isonitrogenous, and to meet or exceed the nutritional requirements of goats [23] (Table 1). The goats received hay *ad libitum* and 600 g of the pelleted feeds per head/day. Animals had free access to water all the time. 

At delivery, three groups of ten male kids born as singletons and homogeneous for live weight (3.50 ± 0.30 kg) were made out from the corresponding goat groups; the kids were reared according to the traditional farming system, suckling only maternal milk *ad libitum* until weaning, which occurred when kids were about 45 days old. After weaning, kids received *ad libitum* the same feed administered to their mothers until they were 60 days old.

Beginning on 9 March 2020, the COVID–19 pandemic state was declared in Italy; therefore, it was not possible to assess growth performances during the trial as well as to collect data at slaughtering and carcass sectioning.

The kids were slaughtered altogether, after fasting for 12 h, according to the official veterinary rules. After 24 h of refrigeration (0–4 °C), the carcasses were split into two halves by the mid-line, and the right side was divided into different cuts (neck, shoulder, leg, steaks, and brisket). Loins were transported from the slaughterhouse to the laboratory under refrigerated conditions for analysis of the *Longissimus lumborum* muscle.

### 2.2. Chemical Composition of Feed

Representative samples of the pelleted feeds were taken every 20 days and mixed to obtain a single final pool for each diet, which was analyzed to determine the chemical composition and fatty acid profile (Table 1). Samples were ground in a hammer mill with a 1-mm screen and analyzed using the following Association of Official Agricultural Chemistry AOAC [24] procedures: dry matter (method 934.01), ether extract (method 920.39), ash (method 942.05), crude protein (method 954.01), crude fiber (method 945.18), ADF and ADL (method 973.18), and amylase-treated neutral detergent fiber (NDF) (method 2002.04). Metabolizable energy was calculated using the INRA system [23].

### 2.3. Chemical Composition and Fatty Acid Profile of Milk

On days 10 and 30 after delivery, milk samples were collected from each goat twice a day (at 7:00 am and 6:00 pm) and pooled together within each group. Milk was stored at 4 °C and immediately transported to the laboratory for the analysis of fat, protein, lactose, and total solids using an infrared milk analyzer (Milkoscan 133-B, Foss Electric, Hillerød, Denmark) previously standardized for goat’s milk. Ash content was detected after burning a sample in a muffle furnace at 550 °C for 5 h.

Total lipids were extracted according to the chloroform/methanol method described by Folch et al. [25]. FAs were methylated using a BF_3_-methanol solution (12% *v*/*v*) [26]. The FA profile was assessed by using a Chrompack CP 9000 gas chromatograph, with a silicate glass capillary column (70% cyanopropyl polysilphenylene-siloxane BPX 70 of SGE Analytical Science, length 50 m, internal diameter 0.22 mm, film thickness 0.25 μm). The temperature program was 135 °C for 7 min, followed by increases of 4 °C per minute up to 210 °C. Fatty acid peaks were identified using a comparative analysis with standard reference mixtures. The fatty acid content was expressed as the percentage of total fatty acid methyl esters (FAME).

### 2.4. Physical Parameters of Muscles

The *Longissimus lumborum* (Ll) muscle was excised from the right half carcass of each kid in order to assess meat color and tenderness. The whole Ll was split into two halves: the proximal one was used for color readings and tenderness assessment, while the second half was used for lipid oxidation, chemical, and fatty acid analysis. 

The color features (L* = Lightness, a* = red index, b* = yellow index) were determined using a Hunter Lab MiniscanTM XE Spectrophotometer (Model 4500/L, 45/0 LAV, 3.20 cm diameter aperture, 10° standard observer, focusing at 25 mm, illuminant D65/10; Hunter Associates Laboratory Inc., Reston, VA, USA). Three readings were taken for each sample by placing the instrument on different meat areas. The instrument was normalized to a standard white tile before performing the analysis (Y = 92.8, x = 0.3162, and y = 0.3322). The reflectance measurements were performed after the samples were allowed to oxygenate in the air for at least 30 min, to take stable measurements [27]. Three samples (1.25 cm in diameter and thickness) of each muscle were tested for tenderness by the Warner-Bratzler Shear (WBS) force system using an Instron 5544 testing machine; samples were assessed in triplicate and perpendicularly sheared in the muscle fiber direction (load cell 50 kg, shearing speed 200 mm/min). Peak force was expressed as kg/cm^2^ [28].

### 2.5. Chemical Composition and Fatty Acid Profile of Kid Meat

In order to analyze the chemical composition of meat, representative sub-samples of the *Longissimus lumborum* muscle from each kid were homogenized [24].

Fat was extracted using a 2:1 chloroform/methanol (*v*/*v*) solution to determine the fatty acid profile [25]. The fatty acids were methylated using a KOH/methanol 2N solution [26] and analyzed by gas chromatography (Shimadzu GC-17A, Shimadzu Corp., Kyoto, Japan) using a silicone-glass capillary column (70% Cyanopropyl Polysilphenylene–siloxane BPX 70, length = 60 m, internal diameter = 0.25 mm, film thickness = 0.25 µm by Thermo Scientific, Waltham, MA USA). The starting temperature was 135 °C for 7 min, and then it was increased by 4 °C/min up to 210 °C. Fatty acids were expressed as a percentage (wt/wt) of total methylated fatty acids.

The Conjugated Linoleic Acid (CLA) content in goat milk and kid meat was assessed as previously described [29].

The food risk factors of meat were determined by calculating the Atherogenic (AI) and Thrombogenic (TI) Indices [30]:

AI = [(C12:0 + 4 × C14:0 + C16:0)] ÷ [ΣMUFA + Σn−6 + Σn−3];
TI = [(C14:0 + C16:0 + C18:0)] ÷ [(0.5 × ΣMUFA + 0.5 × Σn−6 + 3 × Σn−3 + Σn−3)/Σn−6];

where MUFA are monounsaturated fatty acids.

Lipid oxidation was evaluated in *Longissimus lumborum* muscle samples stored at 4 °C for 48 h after slaughtering by measuring the concentration of 2-thiobarbituric acid reactive substances (T-BARS) [31] and expressed as mg malondialdehyde (MDA)/kg meat.

### 2.6. Sensorial Analysis

The sensory attributes of kid meat were assessed according to the procedures described by Pimentel et al. [32]. The *Longissimus lumborum* muscle from the left half carcass of each animal was roasted in a pre-heated oven (170 °C) until the meat temperature was 71 °C at the geometric center of the cut, as recorded by a thermocouple (Hanna Instruments, Villafranca Padovana, PD, Italy). Afterward, fragments of the Ll meat were cut into cubes (2 cm^3^) weighing about 8–10 g and grouped by treatment. 

The consumer test was carried out using forty-one untrained persons, that are habitual consumers of meat (21 men and 20 women, aged 18–72 years). During the sensory evaluation, each taster was provided one meat sample per group without condiments on plastic plates with coded lids containing random three-digit numbers; each taster also received water and crackers for ingestion between tastings to remove residual flavor. Consumer tasters evaluated the following parameters, indicating the intensity of sensation on a 9-point scale: flavor, aroma, softness, juiciness, and overall acceptance. The intensities of the goat meat flavor and aroma characteristics were also evaluated [33].

The analysis was based on five sensory descriptors (Table 2); each descriptor was evaluated using a 9-point semi-structured hedonic scale and continuously anchored at extremities with terms that express intensity.

### 2.7. Statistical Analysis

Data were analyzed using a GLM procedure of SAS software [34] with treatment (diet) as the fixed effect: Y_ij_ = M + A_i_ + E_j_; where Y_ij_ = analyzed trait of meat; M = overall mean; A_i_ = fixed effect of diet; Ej = residual error. 

When the diet effect was significant (*p* < 0.05), means were separated and compared using Tukey’s HSD. Significance was declared at *p* < 0.05; results are reported as least squares mean and standard error of the mean (SEM).

## 3. Results

### 3.1. Chemical and Fatty Acid Composition of Goat Milk

The two diets containing linseed resulted in a significantly (*p* < 0.01) lower content of dry matter in the milk (Table 3). Diet did not significantly affect either the protein or the fat concentration of goat milk, although the linseed + oregano diet determined a lower content of fat.

The fatty acid composition of the goat milk was significantly influenced by the diet. Dietary supplementation with linseed, with or without oregano, significantly increased the concentration of butyric (C4:0; *p* < 0.01) and caproic (C6:0; *p* < 0.05) fatty acids, while it led to a decrease in the concentration of stearic acid (C18:0; *p* < 0.05).

The linseed diet without oregano supplementation lowered (*p* < 0.05) the concentration of several milk fatty acids, such as C20:0, C14:1, and C15:1. The concentration of C20:1 was the lowest in the L + O group (*p* < 0.05). The concentration of total Conjugated Linoleic Acid (CLA) was significantly higher in both of the groups fed linseed as compared to the control (*p* < 0.05). Likewise, the two linseed diets significantly increased (*p* < 0.01) the concentration of C18:3 n3 (α-linolenic) acid.

### 3.2. Kid Meat Physical, Chemical, and Fatty Acid Composition

The loin dissection data are shown in Table 4. The diet did not affect either the loin weight or the percentages of lean, fat, and bone.

Table 5 shows the results concerning meat features from the *Longissimus lumborum* muscle. No significant differences between groups were observed for the pH values of meat, either at slaughtering or after 24 h of refrigeration. None of the meat color parameters were affected by the diet. Meat hardness, assessed by the WBS system, was similar between groups.

While the concentration of malondialdehyde (MDA) was similar between the dietary treatment groups on Day 1 of assessment, after 10 days of storage, meat from the group fed L + O showed the lowest MDA concentration (*p* < 0.01) compared to the other groups, proving that oregano is effective in preserving meat from oxidative processes. 

The chemical composition of the meat is shown in Table 6. The fat content of the kid meat was significantly lowest (*p* < 0.05), following feeding with linseed + oregano, as compared to the diet containing only linseed and to the control. Furthermore, the two diets containing linseed led to a significantly higher (*p* < 0.05) concentration of N-free extracts in the kid meat with respect to the control group.

The fatty acid profile of the kid meat is reported in Table 7. The total concentration of Saturated Fatty Acids (SFAs) was significantly higher (*p* < 0.05) in the control group compared to the L and L + O groups. Among SFAs, greater amounts of myristic (C14:0) and palmitic (C16:0) acids were found in the kid meat from the control group while, in contrast, feeding linseed, with or without oregano, led to a markedly (*p* < 0.05) higher amount of C23:0.

On the other hand, dietary linseed determined a significantly greater (*p* < 0.05) concentration of total Mono Unsaturated Fatty Acids (MUFAs) with respect to the control, especially for C17:1 and C22:1 n-9.

The concentration of total Poly Unsaturated Fatty Acids (PUFAs) was significantly greater (*p* < 0.05) following the diet containing linseed, due to a higher concentration of the individual fatty acids C18:2 n-6, C18:3 n-6, C20:5 n-3, C22:5 n-3, and C22:6 n-3.

The two diets containing linseed, regardless of the presence of oregano, demonstrated a significantly higher (*p* < 0.05) concentration of conjugated linoleic acid (CLA) isomers C18:2_trans10,cis12_ and C18:2_cis9,trans11_, along with the total CLA content, compared with the control diet. 

The diet containing only linseed demonstrated a significantly greater concentration of the total fatty acids of the n-3 (*p* < 0.01) and n-6 (*p* < 0.05) series, along with a lower n-6/n-3 ratio (*p* < 0.05). 

The presence of linseed in the diet, with or without oregano, significantly lowered (*p* < 0.01) the indices of atherogenicity and thrombogenicity of the kid meat compared to the control group.

### 3.3. Sensory Analysis of the Kids’ Longissimus lumborum Muscle 

The results of the sensory analysis of the kid meat from the Ll muscle are shown in Table 8. In this study, feeding with L + O influenced kid meat organoleptic features. Meat from the L + O group was perceived as being more succulent (*p* < 0.05), tender (*p* < 0.01), and juicy (*p* < 0.01) compared to meat from the other two groups. Similarly, the overall acceptability (*p* < 0.05), which expresses the sum of descriptors contributing to the acceptance of the kid meat, was highest following the L + O diet.

## 4. Discussion

This is a preliminary study aimed at enhancing the native Facciuta Lucana goat population reared according to the traditional farming system through dietary supplementation with local and economically sustainable feeds. In order to limit the genetic erosion of native genotypes, it would be desirable to promote interventions that encourage farmers to preserve these populations, which produce milk and meat endowed with interesting nutritional and sensory qualities, that are related to the characteristics of the environment. 

Dietary linseed has been used in sheep [35,36], cows [37], and goats [38] to improve the fatty acid profile of milk; in this study, the diet did not substantially affect the chemical composition of the goat milk, in accordance with previous findings [39,40]. Supplementation with linseed, with or without oregano, significantly increased the concentration of the short-chain saturated fatty acids (SCFA) butyric (C4:0) and caproic (C6:0), as also found by other authors [40,41]. The concentration of palmitic and stearic acids in goat milk decreased following linseed supplementation [36]. In our study, the concentration of total SFAs was lower than that observed in other native goat breeds, showing the effect of the genotype on the fatty acid profile of milk [42].

Interestingly, in our study, the milk content of Conjugated Linoleic Acid (CLA) increased following the linseed diets, in accordance with the findings reported by other authors [38,43]. Total CLA are intermediates of the biohydrogenation of C18:2_cis9,cis12_, which is present in high concentrations in linseed. Many authors have indicated that feeding linseed in different forms leads to an increased proportion of CLA in milk fat following Δ9 desaturase activity in the mammary gland [44]. The concentration of CLA in goat milk in this trial was lower compared to previous research carried out on Ionica goats [45]; CLA content in milk and meat depends on many factors, such as the animal genotype, age, and diet [46,47].

Ruminants are born with underdeveloped rumen and are considered functionally monogastric animals before weaning; during the pre-ruminant stage of life, the animal status is influenced by the mother’s diet [14,16], the feeding methods [19], and the microbial colonization. All these factors may affect meat quality, with particular concern given to the intramuscular fatty acid profile [48].

With regards to the chemical composition of kid meat, the fat content was significantly the lowest following feeding with linseed + oregano, compared to the diet containing only linseed and to the control. This result is in accordance with Rotondi et al. [9] in Garganica suckling kids. 

In this trial, the analysis of the fatty acid profile of kid meat highlighted a higher total concentration of Saturated Fatty Acids (SFAs) in the control group compared to those containing linseed, with or without oregano. Among SFAs, lower amounts of myristic (C14:0) and palmitic (C16:0) acids were found in kid meat from the linseed diets; this is a desirable result from a nutritional point of view, since these fatty acids are held responsible for elevating blood cholesterol [49].

Dietary linseed determined a significantly greater concentration of total MUFAs and PUFAs, and it led to a greater concentration of the total fatty acids of the n-3 and n-6 series, along with a lower n-6/n-3 ratio. In our study, the n-6/n-3 ratio fell within the range 4.58–5.35, which may be considered suitable [50]. In sucking lambs and kids, the n-6/n-3 ratio has been proven to be strongly affected by the breed, the traditional production system, and the environment [51].

Although CLA accounts for a relatively small amount of the total fatty acid composition of foods, it is very important for human health [52,53]. Its main isomer, C18:2 cis-9, trans-11, is effective against cancer and atherogenic diseases and also has positive effects on diabetes and the immune system [54,55]. In our study, the linseed diets determined a significant increase in the CLA content in kid meat, which reflects the difference also found in goat’s milk, in agreement with previous investigations [11,45]. 

Linseed diets lowered the indices of the atherogenicity and thrombogenicity of kid meat compared to the control, in agreement with the results obtained in meat from Jonica kids [11]. These indices provide useful information on the quality of fat, and values below 2.0 are considered appropriate for a healthy diet. 

Feeding strategies aiming to increase the concentration of PUFAs in meat may increase tissue susceptibility to oxidation and perioxidation [9]. In order to limit these processes, several authors have studied the association of plants and herbs having antioxidant properties due to the presence of bioactive compounds (i.e., terpenoids and phenylpropanoids), which are effective in lowering or delaying the occurrence of lipid oxidation [56,57]. Among these, oregano contains carvacrol, thymol, and terpinene, and is considered an alternative to antibiotics due to its antioxidant and antimicrobial properties [58,59].

While the concentration of malondialdehyde (MDA) was similar between the dietary treatment groups on Day 1 of the assessment, after 10 days of storage, meat from the group fed linseed + oregano showed the lowest MDA concentration compared to the other groups, proving that oregano may have been effective in preserving meat from oxidative processes. This may be attributed to oregano’s antioxidant compounds, which are incorporated into muscle cell membranes, thus protecting them against oxidation and increasing meat lipid stability and shelf-life [60].

Several studies have reported that the sensorial properties of goat meat are influenced by the animal’s diet, age at slaughtering, and the farming system [61]. In general, meat from animals fed with concentrate demonstrates higher flavor intensity than those grazed on grass. In this study, feeding with linseed and oregano positively influenced kid meat organoleptic features. Tasters expressed better overall acceptability and appreciated meat for its tenderness, succulence, and juiciness, according to the results obtained from lambs fed extruded linseed in association with oregano [12].

## 5. Conclusions

This paper contributes to a better understanding of meat quality in a local goat population from Basilicata. Due to the small number of individuals of this genotype, further studies are needed to confirm our findings and to strengthen our knowledge of this goat population. 

As expected, the fatty acid profile of the goat milk was affected by the diet, and this also influenced the fatty acid composition of suckling kid meat, which was improved following linseed diets. Furthermore, the addition of oregano to linseed improved overall acceptance by consumers, who appreciated its succulence, tenderness, and juiciness. 

Following these preliminary results, the Basilicata Region decided to include the Facciuta Lucana goat population among the local autochthonous goat breeds to be protected by genetic erosion, thus representing an opportunity for the valorization and promotion of this genotype and its products.

## Figures and Tables

**Table 1:** Ingredients, chemical composition, and fatty acid profile of the diets.

Ingredients (%)	Diet ^1^			Hay
	C	L	L + O	
Corn	31.00	31.00	30.40	
Faba bean	10.00	8.50	8.50	
Wheat bran	10.00	10.00	10.00	
Barley	9.00	9.00	9.00	
Wheat flour shorts	9.00	9.00	9.00	
Sunflower meal	8.00	7.50	7.50	
Dehulled soybean	6.00	6.00	6.00	
Sugar beet pulp	6.00	6.00	6.00	
Soybean hulls	4.00	4.00	4.00	
Extruded linseed	-	3.00	3.00	
Molasses	3.00	3.00	3.00	
Vitamin-mineral premix	3.00	3.00	3.00	
Soybean oil	1.00	-	-	
Oregano	-	-	0.60	
Chemical composition (% on DM basis)				
Crude protein	15.51	15.60	15.61	10.72
Ether extract	3.66	3.70	3.71	1.37
Ash	3.41	3.49	3.56	9.58
Crude fiber	7.91	7.92	8.32	33.94
NDF ^2^	21.19	21.24	21.15	60.38
ADF ^3^	9.58	9.56	9.53	37.43
ADL ^4^	1.79	1.86	1.85	9.31
ME (MJ)	10.16	10.18	10.05	11.25
Fatty acids (% FA methyl esters)				
C16:0 (palmitic)	9.23	7.47	7.39	13.45
C18:0 (stearic)	1.18	3.55	4.08	3.03
C18:1 n-9, cis 9 (oleic)	17.78	18.76	17.99	12.13
C18:2 n-6 (linoleic)	15.16	22.15	20.42	31.00
C18:3 n-3 (α-linolenic)	4.65	31.00	30.68	2.57
C22:5 n-3 (DPA)	0.46	0.17	0.27	0.00
C22:6 n-3 (DHA)	0.29	0.28	0.28	0.01

^1^ C, control feed; L, feed containing 3% extruded linseed; L + O, feed containing 3% extruded linseed + 0.6% oregano. ^2^ NDF, neutral detergent fiber; ^3^ ADF, acid detergent fiber; ^4^ ADL, acid detergent lignin.

**Table 2:** Descriptors used in the quantitative descriptive sensory analysis of kid meat.

Descriptor	Definition
“Kid” flavor ^a^	Mixed experience of olfactory, gustatory, and tactile sensations perceived during the tasting. Flavor intensity of kid meat.
Succulence ^b^	First perception of the quantity of liquid liberated by the sample of meat in the mouth.
Tenderness ^c^	The force required to compress a piece of meat between the molar teeth, evaluated at the first bite.
Juiciness ^d^	Perception of the amount of liquid released from the meat sample in the mouth after the fifth bite.
Overall acceptance ^e^	Sum of quality attributes that will contribute to determine the degree of product acceptance by panelists.

^a^ 0 = Not detected, 9 = very intense; ^b^ 0 = extremely dry, 9 = extremely succulent; ^c^ 0 = very tough, 9 = very tender; ^d^ 0 = very dry, 9 = very juicy; ^e^ 0 = very bad, 9 = very good.

**Table 3:** Chemical and fatty acid composition (%) of goat milk *.

Item	Diet ^1^			SEM ^2^	*p*-Value
	C	L	L + O		
Dry matter	11.67 ^A^	11.00 ^B^	10.09 ^B^	0.336	0.005
Protein	3.57	3.50	3.57	0.116	0.376
Fat	2.66	2.55	1.95	0.325	0.143
Ash	0.77	0.78	0.78	0.0.12	0.829
Lactose	4.67	4.17	3.78	0.350	0.057
C4:0	0.78 ^B^	1.15 ^A^	1.11 ^A^	0.082	0.007
C6:0	1.12 ^b^	1.58 ^a^	1.40 ^a^	1.114	0.014
C8:0	1.80	2.25	2.02	0.166	0.354
SCFA ^3^	3.70	4.98	4.53	0.076	0.214
C10:0 (capric)	8.20	8.35	8.51	0.882	0.526
C11:0	0.06	0.08	0.06	0.014	0.192
C12:0 (lauric)	3.59	3.55	3.32	0.286	0.503
C13:0	0.08	0.09	0.08	0.011	0.294
C14:0 (myristic)	9.38	8.87	9.13	0.431	0.429
C15:0	1.07	0.88	0.96	0.139	0.370
C16:0 (palmitic)	25.19	24.93	24.28	0.716	0.136
C17:0	0.64	0.60	0.66	0.049	0.370
MCFA ^4^	48.21	48.35	46.00	0.074	0.163
C18:0 (stearic)	12.31 ^a^	11.10 ^b^	11.87 ^b^	0.793	0.0296
C20:0	0.46 ^b^	0.69 ^a^	0.78 ^a, b^	0.057	0.016
C21:0	0.18 ^a, b^	0.19 ^a^	0.14 ^b^	0.013	0.023
LCFA ^5^	12.95	10.98	13.79	0.742	0.087
∑ SFA ^6^	64.86	64.37	64.40	1.837	0.812
C14:1	0.35 ^a, b^	0.23 ^b^	0.42 ^a^	0.057	0.035
C15:1	0.29 ^a^	0.22 ^b^	0.28 ^a^	0.019	0.040
C16:1 trans (palmitoleic)	0.27	0.26	0.24	0.022	0.347
C16:1 cis	0.40	0.41	0.32	0.056	0.333
C17:1	0.16	0.16	0.20	0.022	0.282
C18:1 n9 (oleic)	23.55	23.51	23.63	1.392	0.747
C20:1 n9	0.15 ^a^	0.12 ^a, b^	0.06 ^b^	0.03	0.046
∑ MUFA ^7^	25.17	24.91	25.16	1.433	0.902
C18:2 n6 (linoleic)	3.78	3.96	3.23	0.326	0.177
C18:2 _trans10,cis12_	0.05	0.08	0.09	0.007	0.060
C18:2 _cis9,trans11_	0.10	0.12	0.13	0.012	0.664
Total Conjugated Linoleic Acid (CLA)	0.15 ^b^	0.20 ^a^	0.22 ^a^	0.060	0.050
C18:3 n6 (γ-linolenic)	0.51	0.72	0.66	0.090	0.147
C18:3 n3 (α-linolenic)	0.04 ^B^	0.10 ^A^	0.10 ^A^	0.008	0.001
C20:2 n6	0.07	0.07	0.18	0.069	0.247
C20:3 n6	0.04	0.04	0.04	0.002	0.055
∑ PUFA ^8^	4.44	4.59	4.22	0.356	0.493
∑ UFA ^9^	29.61	29.48	29.46	1.683	0.945

* Results are reported as least square means; ^1^ C, control feed; L, feed containing 3% extruded linseed; L + O, feed containing 3% extruded linseed + 0,6% oregano; ^2^ SEM: Standard error of means; ^3^ SCFA: Short Chain Fatty Acids; ^4^ MCFA: Medium Chain Fatty Acids; ^5^ LCFA: Long Chain Fatty Acids; ^6^ SFA: Saturated Fatty Acids (sum of C4:0 + C6:0 + C8:0 + C10:0 + C12:0 + C13:0 + C14:0 + C15:0 + C16:0 + C17:0 + C18:0 + C20:0 + C21:0); ^7^ MUFA: Mono Unsaturated Fatty Acids (sum of C14:1 + C15:1 + C16:1 c + C16:1 t + C17:1 + C18:1 n9 + C20:1 n9); ^8^ PUFA: Poly Unsaturated Fatty Acids (sum of C18:2 n6 + C18:2 c12;t10 + C18:2 c9;t11 + C18:3 n6 + C18:3 n3 + C20:2 n6 + C20:3 n6); ^9^ UFA: Unsaturated Fatty Acids (sum of MUFA + PUFA). Means with different letters within each row significantly differ: ^a, b^: *p* < 0.05; ^A, B^: *p* < 0.01.

**Table 4:** Dissection data (% on weight) of the loin in kids *.

Item	Diet ^1^			SEM ^2^	*p*-Value
	C	L	L + O		
Loin weight (g)	219.50	209.73	220.34	9.588	0.449
Lean (%)	48.46	48.37	49.65	5.207	0.301
Dissectible fat (%)	11.71	9.94	10.30	0.503	0.204
Bone (%)	39.83	41.69	10.05	0.509	0.112

* Results are reported as least squares mean; ^1^ C, control feed; L, feed containing 3% extruded linseed; L + O, feed containing 3% extruded linseed + 0.6% oregano. ^2^ SEM: Standard error of means.

**Table 5:** Meat characteristics from the *Longissimus lumborum* muscle * in kids.

Item	Diet ^1^			SEM ^2^	*p*-Value
	C	L	L + O		
pH_1_ (pH at 1 h *post-mortem*)	6.53	6.63	6.64	0.136	0.054
pH_24_ (pH at 24 h *post-mortem*)	5.81	5.83	5.74	0.134	0.059
L * (Lightness)	47.75	50.04	47.39	1.019	0.112
a * (Red index)	4.26	4.08	4.45	0.753	0.753
b * (Yellow index)	11.20	11.68	11.14	0.176	0.101
WBS ^3^ (kg/cm^2^)	4.90	4.80	5.09	0.540	0.537
MDA ^4^ (mg/kg meat), Day 1	0.06	0.06	0.05	0.006	0.107
MDA ^4^ (mg/kg meat), Day 10	0.36 ^A^	0.30 ^A^	0.13 ^B^	0.014	0.001

* Results are reported as least squares mean; ^1^ C, control feed; L, control feed + 3% extruded linseed; L + O, control feed + 3% extruded linseed + 0.6% oregano; ^2^ SEM: Standard error of means; ^3^ WBS, Warner–Bratzler shear force; ^4^ MDA, malondialdehyde. Means with different letters within each row significantly differ: ^A, B^: *p* < 0.01.

**Table 6:** Chemical composition (%) of the *Longissimus lumborum* muscle * in kids.

Item	Diet ^1^			SEM ^2^	*p*-Value
	C	L	L + O		
Moisture	72.70	72.71	74.53	1.808	0.497
Protein	20.01	20.45	19.38	1.094	0.502
Lipid	5.07 ^a^	3.82 ^b^	3.37 ^c^	0.674	0.049
Ash	2.01	2.58	2.30	0.267	0.170
N free-extract	0.21 ^b^	0.44 ^a^	0.42 ^a^	0.185	0.035

* Results are reported as least squares mean; ^1^ C, control feed; L, feed containing 3% extruded linseed; L + O, feed containing 3% extruded linseed + 0.6% oregano. ^2^ SEM: Standard error of means. Means with different letters within each row significantly differ: ^a, b, c^: *p* < 0.05.

**Table 7:** Fatty acid composition (% total FAME) of the *Longissimus lumborum* muscle * in kids.

Item	Diet ^1^			SEM ^2^	*p*-Value
	C	L	L + O		
C10:0 (capric)	0.20	0.17	0.15	0.029	0.214
C12:0 (lauric)	0.73	0.55	0.57	0.085	0.151
C14:0 (myristic)	8.14 ^a^	6.27 ^b^	7.03 ^b^	0.448	0.012
C15:0	0.66	0.53	0.61	0.043	0.063
C16:0 (palmitic)	26.73 ^a^	23.62 ^b^	24.49 ^b^	0.820	0.020
C17:0	1.00	0.91	0.98	0.062	0.319
C18:0 (stearic)	14.44	14.64	14.80	0.622	0.691
C20:0	0.07	0.09	0.12	0.017	0.077
C21:0	0.04	0.05	0.03	0.004	0.056
C22:0	0.16	0.22	0.18	0.021	0.079
C23:0	0.28 ^b^	0.48 ^a^	0.35 ^a^	0.051	0.011
∑ SFA	52.45 ^a^	47.53 ^b^	49.31 ^b^	1.157	0.011
C14:1	0.14	0.12	0.19	0.024	0.069
C15:1	0.25	0.22	0.24	0.023	0.401
C16:1 *trans* (palmitoleic)	0.41	0.35	0.38	0.023	0.102
C16:1 *cis*	1.52	1.36	1.68	0.146	0.153
C17:1	0.28 ^b^	0.47 ^a^	0.48 ^a^	0.052	0.018
C18:1 n-9 *trans* (elaidic)	0.17	0.15	0.17	0.011	0.092
C18:1 n-9 *cis* (oleic)	31.05	31.08	32.68	0.667	0.109
C20:1 n-9	0.12	0.12	0.25	0.064	0.165
C22:1 n-9	1.63 ^b^	2.95 ^a^	2.00 ^a^	0.360	0.024
∑ MUFA	35.57 ^b^	36.82 ^a^	38.07 ^a^	0.584	0.013
C18:2 n-6 *trans*	0.16	0.48	0.17	0.128	0.102
C18:2 n-6 *cis* (linoleic)	5.00 ^b^	6.75 ^a^	5.87 ^b^	0.504	0.022
C18:2 trans10,cis12	0.02 ^b^	0.05 ^a^	0.06 ^a^	0.001	0.048
C18:2 cis9,trans11	0.05 ^b^	0.08 ^a^	0.10 ^a^	0.003	0.047
Total Conjugated Linoleic Acid (CLA)	0.07 ^b^	0.13 ^a^	0.16 ^a^	0.020	0.049
C18:3 n-6 (γ-linolenic)	0.40 ^B^	0.70 ^Aa^	0.55 ^b^	0.048	0.001
C18:3 n-3 (α-linolenic)	0.13	0.13	0.12	0.007	0.110
C20:2 n-6	0.33	0.50	0.36	0.057	0.069
C20:4 n-6 (ARA)	0.06	0.09	0.06	0.011	0.088
C20:5 n-3 (EPA)	0.15 ^a, b^	0.21 ^a^	0.13 ^b^	0.020	0.029
C22:5 n-3 (DPA)	0.67 ^B^	1.13 ^Aa^	0.81 ^b^	0.102	0.008
C22:6 n-3 (DHA)	0.19 ^B^	0.39 ^A^	0.25 ^B^	0.032	0.001
∑ PUFA	7.16 ^b^	10.51 ^a^	8.48 ^b^	0.792	0.018
∑ UFA	42.73 ^b^	47.33 ^a^	46.55 ^a^	1.600	0.015
Other acids	4.82	5.14	4.15	1.446	0.417
n-3	1.14 ^B^	1.86 ^A^	1.31 ^B^	0.270	0.002
n-6	5.95 ^b^	8.52 ^a^	7.01 ^b^	2.379	0.020
n-6/n-3	5.22 ^a^	4.58 ^b^	5.35 ^a^	1.624	0.047
A.I.	1.54 ^A^	1.15 ^B^	1.38 ^B^	0.074	0.003
T.I.	2.23 ^A^	1.86 ^B^	2.00 ^B^	0.107	0.003

* Results are reported as least squares mean; ^1^ C, control feed; L, feed containing 3% extruded linseed; L + O, feed containing 3% extruded linseed + 0.6% oregano. ^2^ SEM: Standard error of means. SFA—saturated fatty acids (sum of C10:0 + C12:0 + C14:0 + C15:0 + C16:0 + C17:0 + C18:0 + C21:0 + C22:0 +C24:0); MUFA—monounsaturated fatty acids (sum of C14:1 + C15:1 + C16:1 c + C16:1 t + C17:1 + C18:1 n-9 t + C18:1 n-9 c + C20:1 n-9 + C22:1 n-9); Total n-6 (sum of C18:2 n-6 t + C18:2 n-6 c + C18:2 c12;t10 + C18:2 c9;t11 + CLA + C18:3 n-6 + C20:2 n-6 + C20:4 n-6); Total n-3 (sum of C18:3 n-3 + C20:5 n-3 + C22:5 n-3 + C22:6 n-3); PUFA—polyunsaturated fatty acids (sum of n-6 + n-3); means with different letters within each row significantly differ: ^a, b^: *p* < 0.05; ^A, B^: *p* < 0.01.

**Table 8:** Consumer appeal of meat from the *Longissimus lumborum* muscle in kids *.

Item	Dietary Treatment ^1^			SEM ^2^	*p*-Value
	C	L	L + O		
Tasters (n.)	41	41	41		
Flavor	6.07	5.61	5.78	2.473	0.068
Succulence	6.46 ^b^	6.50 ^b^	7.22 ^a^	1.248	0.044
Tenderness	6.63 ^B^	6.68 ^B^	7.54 ^A^	1.388	0.007
Juiciness	4.23 ^B^	4.39 ^B^	5.60 ^A^	1.426	0.006
Overall acceptance	8.41 ^b^	8.10 ^b^	8.71 ^a^	1.317	0.038

* Results are reported as least squares mean; ^1^ C, control feed; L, feed containing 3% extruded linseed; L + O, feed containing 3% extruded linseed + 0.6% oregano. ^2^ SEM: Standard error of means. Means with different letters within each row significantly differ: ^a, b^: *p* < 0.05; ^A, B^: *p* < 0.01.

## Data Availability

All data generated or analyzed during this study are included in this article.

## Supplementary Materials

The following supporting information can be downloaded at: https://www.mdpi.com/article/10.3390/ani13193050/s1, Table S1: Description of the “Facciuta Lucana” authocthonous goat population. Refs in Supplementary Materials [62,63,64].
