# Supplementary material for: Dietary Supplementation with Oregano and Linseed in Autochthonous “Facciuta Lucana” Goats: Effects on Meat Quality Traits in Suckling Kids
Source: Animals (Basel). 2023 Sep 28;13(19):3050. doi: 10.3390/ani13193050 (PMC10571778; doi:10.3390/ani13193050)
Supplement: Supplementary file 1 [file animals-13-03050-s001.zip › animals-2576541-supplementary.pdf]

Table S1. Description of the "Facciuta Lucana" authocthonous goat population.

The Facciuta Lucana is a local goat population from Basilicata (also known as Lucania, South Italy). It is thought to belong to the Mediterranean group of goat populations. Recent genetic studies performed using a commercial medium-density SNP (Single Nucleotide Polymorphism) array have highlighted genetic closeness between this population and goat breeds from Central and Southern Italy, possibly because of a common ancient origin and/or recurrent gene flow among neighbouring geographic areas [1-3].

Adult goats have a medium-large body size, a big, triangular, elongated and well-proportioned head with medium-large and semi-upright ears. Both females and males present flat divergent horns which show a lyre-like shape and that can reach the length of 30 cm in goats and 50 cm in bucks. Some animals are lack of horns. A beard may be present in both sexes as well as a tuft of shaggy hair in the frontal area.

The coat generally has a tan, black colour with long hairs and reddish reflections, while the distal part of the limbs, the belly and the perianal area may be white or light beige. The face profile can be straight, or slightly hilly, and two light coloured stripes (named "*frisature*" in Italian) run along the face, sideways and parallel to the nose, until reaching the eye area. These stripes are also observed in other local goat breeds from Central/Southern Italy such as Nicastrese, Capestrina, Valfortorina and Teramana, as well as in goat breeds from other countries.

The length of the trunk is considerable, the thorax and abdomen are large, and the dorsal line is straight. The limbs are long and robust and well adapted to graze on bushes and wooded environments. Some biometric measurements of the Facciuta Lucana adult males and females are reported in Table 1.

Table 1. Biometric features of the Facciuta Lucana goat breed in relation to sex.

	Adult male	Adult female
Weight (kg)	50–74	41–64
Height at withers (cm)	70–82	64–73
Chest height (cm)	25–36	24–32
Chest width (cm)	13–19	14–18
Trunk length (cm)	41–50	32–45

The Facciuta Lucana goat is bred with traditional grazing systems, with housing in the evening and during cold and rainy winter days. The average age at first delivery is about 12 months; the prolificity is high, so trigeminal offspring is frequent. Some productive and reproductive features are reported in Table 2.

Table S2. Some productive and reproductive performances of the Facciuta Lucana goat population.

Milk yield and composition:	
Milk daily average production (litres/day/head)	2.5–3.0
Lactation average length (days)	240–250
Total milk yield/lactation (litres)	600
Dry matter (%)	16.02
Protein (%)	5.70
Fat (%)	6.15
Lactose (%)	4.17
Cheese yield:	
for cacioricotta production (%)	15–17
for fresh cheese production (%)	20–22
Reproductive parameters:	
Age at first service (days)	240
Gestation period (days)	155
Fertility (% N. of goats kidding/N. of goats mated)	95.2
Prolificacy (% N. of kids born/N. of goats kidding)	175
Litter size (N. of kids born/goat)	2.58

Nowadays, the population size is very limited and, to best of our knowledge, it includes 95 animals, out of which 60 adult females, 5 adult males and 30 replacement females (Figure 1).

Recently, the Italian Ministry of Agriculture and Food Sovereignty has included the "Facciuta Lucana" goat population in the national registry of endangered species.

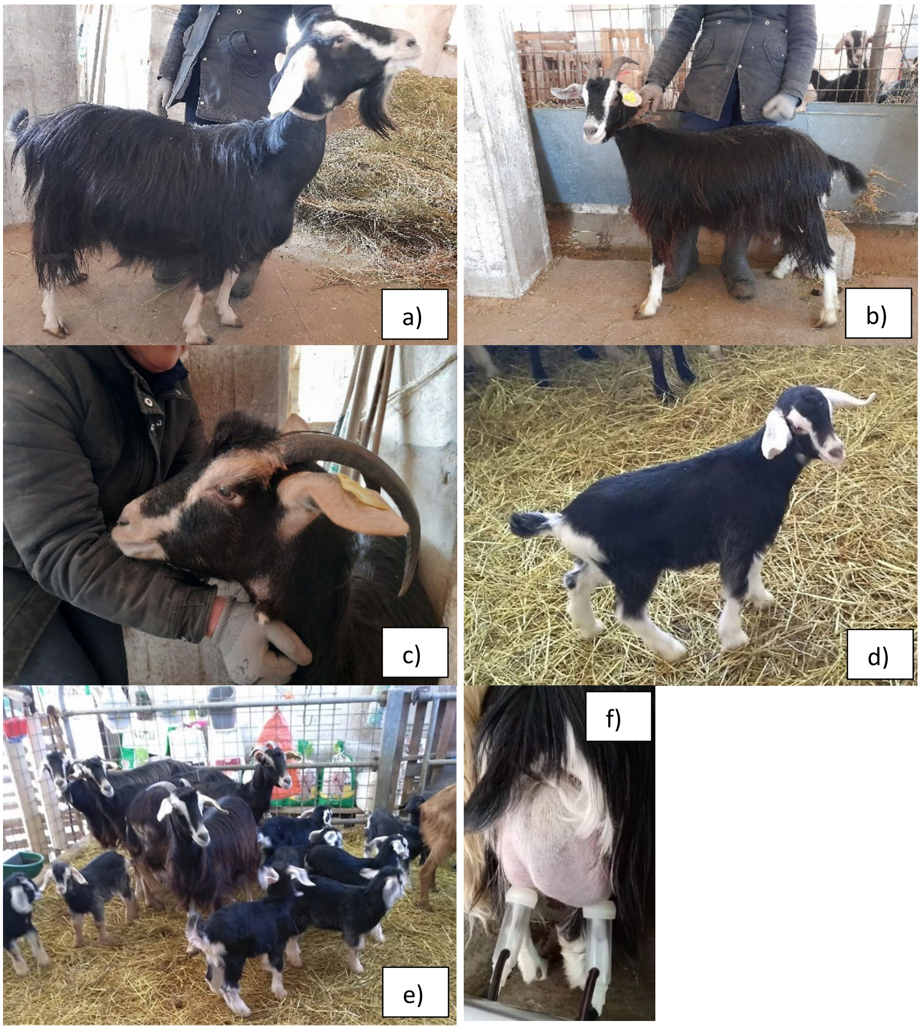

Figure S1. Some specimens of the Facciuta Lucana goat breed: a) adult dam; b) young male; c) ancestor buck; d) kid; e) group of kids with their dams; f) detail of lactating dam.

<u>References</u>

1) Landi, V.; Tarricone, S., Ragni, M.; Giannico, F.; Marsico, G.; Sarti, F.M.; Lasagna, E., Ceccobelli, S.; Crepaldi, P.; Pilla, F.; Ciani, E. Selection signatures in Italian goat breeds sharing the "facciuto" phenotype. *Book of abstract of International Conference on "Management of animal and plant genetic resources"*. Tirana, Albania, November 19th 2021.
2) Tarricone, S.; Landi, V.; Ragni, M.; Giannico, F.; Rillo, L.; Matassino, D.; Crepaldi, P.; Marsico, G.; Ciani, E. The "Murciunara" goat population from Southern Italy: insights into its possible genetic origin. *Book of abstract of International Conference on "Management of animal and plant genetic resources"*. Tirana, Albania, November 19th 2021.
3) Landi, V.; Tarricone, S.; Ragni, M.; Giannico, F.; Marsico, G.; Sarti, F.M.; Lasagna, E.; Ceccobelli, S.; Crepaldi, P.; Pilla, F.; Ciani, E. Segnali di selezione per il fenotipo facciuto in alcune razze caprine italiane. Biodiversità 2021: Agricoltura, ambiente e salute: XIII Convegno Nazionale sulla Biodiversità, Foggia, 7-9 settembre 2021. ISBN: 9788874271016.
